# Improving the Safety and Security of Those Engaged in Global Health Traveling Abroad

**DOI:** 10.9745/GHSP-D-16-00203

**Published:** 2016-12-23

**Authors:** Ranit Mishori, Andrew Eastman, Jessica Evert

**Affiliations:** a Georgetown University School of Medicine, Global Health Initiatives, Department of Family Medicine, Washington, DC, USA.; b Georgetown University/Providence Hospital Family Medicine Residency Program, Washington, DC, USA.; c University of California, San Francisco, Child Family Health International, San Francisco, CA, USA.

## Abstract

We need to improve the safety and security of global health students, faculty, residents, and workers who travel abroad, particularly those affiliated with smaller organizations or educational programs that lack resources and protocols. We offer a checklist covering 6 core elements: (1) institutional commitment, (2) trainee and faculty participation, (3) safety and security assessment and analysis, (4) risk and hazard prevention, (5) safety training, and (6) program evaluation.

## INTRODUCTION

Thousands of students, residents, faculty, and other professionals travel around the world every year to work on global health issues—some for short-term assignments, conferences, or workshops and others for extended periods. Many of them travel through established organizations, programs, and universities that have existing resources dedicated to risk management or protocols for safety and security. This is often not the case, however, for learners and faculty from independent programs or higher-education organizations, or for those linked to post-graduate programs in the medical field—namely residency programs. Most residency programs are affiliated with universities or medical schools that have experience providing resources for global travelers or have well-established academic partners and sites internationally. Many other programs, however, are independent and loosely affiliated, or they are linked to smaller institutions, community-based hospitals, faith-based organizations, and hospital systems that lack institutional risk management experience, partnerships, and resources. Even among those programs and institutions who understand and value the need to properly prepare travelers for work in other countries, their plans for safety, security, and risk management may be lax or even absent.^1^^,^^2^ The critical need to safeguard the security of the global health trainees and practitioners, particularly those who do not have institutional backing, is falling through the cracks.

Many global health-related programs lack institutional risk management experience, partnerships, and resources to properly prepare travelers for work in other countries.

## RISKS AND CHALLENGES

Students, residents, and faculty who travel for global health-related work—whether for clinical care, public health, research, development, training, or other purposes—may be exposed to various risks including infectious diseases,^3^^–^^6^ road traffic accidents and injuries,^7^^–^^9^ conflict-related violence, and deliberate attacks. Violent attacks are of particular concern because prevention and management require considerable institutional planning and resources—but it is also an area that often lacks forethought and attention. For those with mental health issues, the stress of travel itself, combined with the pressures of separation from known environments, cultural differences and expectations, and adjustment to different housing and the local environment, may exacerbate existing conditions.^10^

### Risks to Personal Safety in the Context of Violence

Violence against foreigners (e.g., petty robbery, sexual violence, and carjacking) has always been a concern. Some health care professionals believe that their profession shields them from attacks, but they may be upset to know that attacks *specifically* targeting health care providers are increasing, particularly in conflict zones, but also in post-conflict and non-conflict zones.^11^^–^^15^ Acts of violence targeting health care providers violate international humanitarian laws^16^ and have caused innumerable losses, prompting an outcry among those who believe in medical neutrality.^17^^–^^19^ Between 2012 and 2014, of all armed violence incidents against the health care system, the majority of the targets were health care facilities, followed by medical transports.^20^

Attacks *specifically* targeting health care providers are increasing, particularly in conflict zones, but also in post-conflict and non-conflict zones.

Increasingly, the primary motivation of many violent attacks is political. One study estimated a 55% increase in politically motivated attacks between 2004 and 2008.^21^ The authors of the study concluded that "even though many organizations make considerable efforts to dissociate themselves from political actors and project an image of neutrality … organizations are being attacked not just because they are perceived to be cooperating with Western political actors, but because they are perceived as wholly a part of the Western agenda."^21^

In Mali and Burkina Faso in late 2015 and early 2016, al Qaeda terrorists killed nearly 50 people from various parts of the world in a series of coordinated attacks targeting hotels frequented by businesspeople and foreigners, and injured many more. These attacks brought to light the potential dangers to national and international health and development workers regardless of the purpose of their visit or work. These kinds of attacks are worrisome even to the most experienced and savvy global health professionals.^22^^,^^23^ No longer can those engaged in global health work expect to stay safe even in locations outside of direct service sites such as hotels, malls, and conference centers. Some major international organizations have updated safety security protocols and now require training for their staff.^23^

Personal safety is an integral component of guidelines created by the Working Group on Ethics Guidelines for Global Health Training to introduce trainees and others involved in global health to ethical issues that may arise during short-term training experiences abroad.^24^ The Association of Medical Education in Europe also addresses the issue of personal safety among its undergraduate medical students, acknowledging the crucial importance of risk management for those students who study internationally.^25^ These guidelines, however, do not include safety and security measures in the context of conflict, political, or terrorism-related violence. The U.S. Peace Corps requires all Peace Corps Volunteers to participate in up to 12 weeks of intensive preservice training in their country of service, which covers risks associated with serving abroad, coping with unwanted attention, promoting country-specific strategies and best practices to manage risks, and identifying emergency plans.^26^

### Insufficient Resources and Barriers to Improvement

Some smaller programs with partial or no security risk management systems have access to resources through membership organizations, such as InterAction's minimum operating security standards and training course for security management.^27^ However, many students, staff, and faculty engaged in global health do not have the backing of international health and development organizations, or even universities, with established safety and security planning, protocols, and dedicated staff.

Smaller organizations face many barriers in improving preparedness for safety and security, such as lack of capacity and funding.

Common barriers that smaller organizations face in improving preparedness for safety and security may include:

- General lack of awareness of safety and security threats depending on the region, political stability, practice setting, type of work expected, and residential setting
- No independently developed institutional safety and security protocols
- Lack of capacity to develop and manage safety and security programs
- Lack of funding to create a training and support infrastructure
- Lack of information on the effectiveness of existing security training
- Lack of funding for security training by outside or independent companies that specialize in security training
- Lack of knowledge of existing standardized protocols used by other organizations and programs, such as InterAction's minimum operating security standards^28^
- No access to credible and timely security risk and threat assessment
- Lack of guidance about whether individuals with certain profiles or characteristics (health, or otherwise) should be advised not to go to certain locations.

### Lack of Pre-Departure Training

Few published articles exist that document safety and security training as part of pre-departure preparations for students, residents, or faculty involved in global health work abroad.^29^^–^^31^ Even fewer studies document whether programs have any impact on mitigation of adverse events. One study concluded that "medical students are often poorly prepared for the … safety dilemmas they encounter during these electives" based on a survey of 23 medical students returning from international study.^32^ Another study, focusing on accidents among medical students studying abroad, mentioned that there is often a lack of advice on personal safety issues.^33^ A Canadian study of medical school faculty also noted gaps in pre-departure planning and concluded that reviews of health and safety should be mandatory for all international electives.^34^ A few studies have documented student satisfaction with health and safety training, but they lack details about what safety training their programs covered.^35^^,^^36^

### Gender Differences

Some studies have noted gender differences in security risks and training. For example, a study using the Security in Numbers Database looked at the risk profile of 1,361 staff members affected in 615 security incidents and found that women were targeted more frequently for petty crime and sexual assault.^37^ Men, however, were more likely to be involved in violent encounters—and more likely to be killed or injured.^38^ Another study of security perceptions among NGO workers around the world found that a greater proportion of men than women received security training.^39^

## WHAT CAN BE DONE

Global health institutions and programs of all types and sizes can plan more carefully for the safety and security of students, residents, and faculty with the help of existing resources and the guidance provided in this article. We aim to offer a way forward for sponsor organizations (that is, the institutions and programs that send travelers abroad), with specific actions to take and questions to ask to increase awareness and prompt conversations about this topic, while recognizing that host (in-country) organizations also bear responsibility for risk management.

Global health institutions and programs of all types and sizes can plan more carefully for the safety and security of students, residents, and faculty with the help of existing resources and the guidance provided in this article.

According to the United Nations Office for Disaster Risk Reduction (UNISDR), being fully prepared includes "a sound analysis of disaster risks and good linkages with early warning systems, and includes such activities as contingency planning, stockpiling of equipment and supplies, the development of arrangements for coordination, evacuation and public information, and associated training and field exercises. These must be supported by formal institutional, legal, and budgetary capacities."^40^ The following sections cover some of the aspects of being fully prepared to send students and faculty on global health rotations.

### Emergency Management Planning

When planning for emergency management, it is helpful to consider the following 4 phases^40^^,^^41^:

1. **Mitigation:** refers to activities that may help prevent or reduce the chances of an emergency from happening, or to reduce the harmful effects of unavoidable emergencies
2. **Preparedness:** consists of strengthening individual and institutional capacities to effectively anticipate, respond to, and recover from hazardous events or conditions
3. **Response:** details what actions to take during an emergency to save lives, reduce health impacts, ensure public safety and basic needs, and prevent further harm or damage
4. **Recovery:** refers to activities carried out after the emergency to return to a safer environment or to normal operations

Organizations should consider each of the 4 phases as they create their overall safety and security plans, including strategies and policies as well as individual and institutional responsibilities—from what the ultimate individual beneficiary (student, resident, faculty member) should know, to what protocol should be developed by organizations and programs, to setting national or specialty- and discipline-specific standards for security and safety and sharing resources among various stakeholders.

Organizations should consider each of the following 4 phases in their emergency management planning: (1) mitigation, (2) preparedness, (3) response, and (4) recovery.

### Specific Actions to Take at All Levels

To overcome some of the common barriers faced by organizations, we propose a checklist of specific actions for institutions and individuals at all levels (Table). The list of actions is divided into 6 core elements: (1) institutional commitment, (2) trainee and faculty participation, (3) safety and security assessment and analysis, (4) risk and hazard prevention, (5) safety training, and (6) program evaluation. These actions can serve as a conversation starter for those engaged in global health programming and can jumpstart the process of developing a comprehensive safety and security plan.

**TABLE.** Checklist of Specific Actions for Developing Safety and Security Plans

Core Element	Suggested Actions
Institutional commitment	Ensure that safety and security are institutional priorities Identify a safety and security champion or team and an institutional liaison Create a mission statement related to security, specifically related to the institution's commitment to the safety and security of the students and staff Establish goals and objectives for the global health program Identify what resources and institutional support are needed to ensure the security of staff and students Create open communication avenues with leadership on security and safety during international travel

Trainee and faculty participation	Secure and mandate full participation from all trainees and faculty Establish a culture of expecting safety and security Consider requiring participants to sign safety and security pledges

Safety and security assessment and analysis	Create processes and procedures to continuously monitor and evaluate risks and assess threats at destination sites Conduct an initial assessment of safety and security at destination sites and update these assessments regularly Create incident reporting protocols Create databases for reporting incidents (e.g., injuries, accidents, incidents and near misses, police reports, daily logs) Consider identifying trainee and faculty safety and security profiles (e.g., related medical, cultural, and psychological profiles) and an algorithm for matching individuals to appropriate destination, training, and work sites

Risk and hazard prevention	Create processes and programs to mitigate and control known hazards (e.g., physical changes to compounds such as gates, fences, barriers, window bars, and improved lighting) Create and implement communication response and recovery procedures and protocols Assess what supplies and kits are needed at various work locations, lodgings, and health or medical work sites Identify partners that provide travel insurance and evacuation services Ensure travelers have received appropriate immunizations and required medications Inform travelers about what to do if they become ill at the destination site Work with the local organizations where faculty and students are placed to ensure they also have safety and security plans in place Ensure that trainees and faculty have received appropriate immunizations and have all recommended medications and medical supplies (e.g. malaria prophylaxis, for travelers' diarrhea, HIV PEP-kits, gloves, syringes, etc.)

Safety training	Provide pre-departure travel safety and security training to all staff (whether they travel or not) involved, in classrooms, hands-on workshops, or online Consider having access to trauma-informed care for returning travelers who may need it Provide training in risk recognition and control, and what to do in an emergency Provide a written safety and security plan to all travelers that includes all policies and procedures Make sure security policies address country-specific issues as well as problems that may arise among team members, such as sexual assault Provide frequent opportunities to discuss safety and security concerns, practice skills, and demonstrate competency

Program evaluation	Create mechanisms for recordkeeping and accurate logging of injuries, illnesses, fatalities, incidents, assaults, hazards, corrective actions, interventions, and training Create protocols for regular assessment of incident severity, and identify trends, patterns, and methods of addressing incidents Continuously monitor and modify methods of risk assessment, intervention, and training needs to identify deficiencies and opportunities for improvement Design surveys and post-travel debriefing for all returning staff Create success measures and outcomes and work on tracking successful implementation If feasible, request outside consultation from law enforcement or safety and security experts Beyond checking whether safety and security training programs exist, assess the quality and effectiveness of the programs

We propose a list of specific actions for institutions and individuals at all levels to help jumpstart the process of developing a comprehensive safety and security plan.

### Individual Responsibility

Travelers themselves must be prepared before departure, understand the risks, plan for emergencies, and understand their roles and responsibilities in emergencies. It is the duty of supervisors and program directors to ensure that students, residents, and faculty are empowered to ask questions—and that host institutions (e.g., schools, agencies, programs, facilities) are prepared to answer them.

Questions that prospective travelers should consider asking their host *and* sponsoring institution:

- What are you doing to enhance my safety and security?
- What are the threats I may face where I am going?
- What safety and security questions should I ask?
- How do I find out if now is the right time to go?
- What should I look out for (regarding safety and security protocols) when selecting a small organization to travel with?
- What should I look for in regard to food and lodging?
- What are the communication procedures should problems arise?
- Are there added services and costs for enhanced safety measures?
- Should I engage in safety and security training with an outside group?
- Should I purchase travelers insurance?
- Does my health insurance cover conditions encountered abroad?
- Should I purchase security evacuation insurance? Are there protocols for medical evacuation?
- What security responsibilities will be required of me?
- What incidents have you had in the past and how did you handle them?
- What safety and security protocols are in place in case of an unexpected event?

The U.S. Department of State issues travel warnings and alerts online to assist travelers with travel plans to any country in the world (see https://travel.state.gov/content/passports/en/alertswarnings.html). For example, travel warnings are issued when there are unstable government conditions, civil war, ongoing intense crime or violence, or terrorist attacks, while travel alerts are for short-term events such as election seasons when strikes and demonstrations are likely to happen and health alerts such as disease outbreaks. It also hosts a free service called "Smart Traveler Enrollment Program" (STEP) that allows U.S. citizens and nationals traveling abroad to enroll their trip with the nearest U.S. Embassy or Consulate (see https://step.state.gov/step/). The service provides travelers important information about safety conditions in the destination country and helps the U.S. Embassy, family, and friends contact the traveler in an emergency.

The U.S. Centers for Disease Control and Prevention provides a host of travel resources on its website, including information about vaccinations (see http://wwwnc.cdc.gov/travel/page/resources-for-travelers).

## CONCLUSION

As the number of students, residents, and faculty engaged in global health grows, we need to promote a serious dialogue on the topic of safety and security abroad. We must exchange ideas and best practices, conduct joint research, and learn from each other and from organizations with a proven track record of ensuring the safety and security of their staff.

A recent webinar by the Consortium of Universities for Global Health addressed some of these issues and is a welcome step in the right direction (http://www.cugh.org/events/rules-road-global-health-safety-and-security-deploying-students-staff-and-clinicians-overseas). However, more needs to be done. We urge global health program directors of all disciplines and specialties to take this issue very seriously. We encourage all health profession education organizations, specialty societies, and global health organizations to start synchronizing preparedness efforts and create unified protocols, manuals, checklists, standardized security procedures, or best practices and share them freely with those who lack the capacity to create their own.

We also call on global health and health profession education conference organizers to make this issue a routine part of their call for proposals. Additionally, researchers and scholars should be encouraged to collect and publish case reports, develop best practice recommendations, and design studies looking at the effectiveness of safety plans and security training for the global health workforce at large.

## Notes

**Peer Reviewed**

## References

[1] Melby MK Loh LC Evert J Prater C Lin H Khan OA Beyond medical missions to impact-driven short-term experiences in global health (STEGHs): ethical principles to optimize community benefit and learner experience. Acad Med. 2016;91(5):633–638. 10.1097/ACM.0000000000001009. 26630608

[2] Imperato PJ Bruno DM Monica Sweeney M Ensuring the health, safety and preparedness of U.S. medical students participating in global health electives overseas. J Community Health. 2016;41(2):442–450. 10.1007/s10900-016-0169-7. 26882901

[3] Nasci RS Wirtz RA Brogdon WG Protection against mosquitoes, ticks, & other arthropods. In: Centers for Disease Control and Prevention. Health information for international travel 2016. New York: Oxford University Press; 2016 Available from: http://wwwnc.cdc.gov/travel/yellowbook/2016/the-pre-travel-consultation/protection-against-mosquitoes-ticks-other-arthropods

[4] Backer HD Water disinfection for travelers. In: Centers for Disease Control and Prevention. Health information for international travel 2016. New York: Oxford University Press; 2016 Available from: http://wwwnc.cdc.gov/travel/yellowbook/2016/the-pre-travel-consultation/water-disinfection-for-travelers

[5] Küpper T Rieke B Neppach K Morrison A Martin J Health hazards and medical treatment of volunteers aged 18–30 years working in international social projects of non-governmental organizations (NGO). Travel Med Infect Dis. 2014;12(4):385–395. 10.1016/j.tmaid.2013.11.004. 24332435

[6] Wyler N Green S Boddington N Davies C Friedli K Lankester T Travel related illness in short-term volunteers from the UK to developing countries. Travel Med Infect Dis. 2012;10(4):172–178. 10.1016/j.tmaid.2012.04.002. 22647772

[7] Sleet DA Ederer DJ Ballesteros MF Injury prevention. In: Centers for Disease Control and Prevention. Health information for international travel 2016. New York: Oxford University Press; 2016 Available from: http://wwwnc.cdc.gov/travel/yellowbook/2016/the-pre-travel-consultation/injury-prevention

[8] Brinker K Head CA Johnson CY Funk RH Injuries and illnesses among American Red Cross responders–United States, 2008–2012. Disaster Med Public Health Prep. 2014;8(5):404–410. 10.1017/dmp.2014.99. 25351536

[9] Nurthen NM Jung P Fatalities in the Peace Corps: a retrospective study, 1984 to 2003. J Travel Med. 2008;15(2):95–101. 10.1111/j.1708-8305.2008.00185.x. 18346242

[10] McCabe L Mental health and study abroad: responding to the concern. Int Educator. 2005:14(6):52–57. Available from: https://www.nafsa.org/_/File/_/InternationalEducator/EducationAbroadNovDec05.pdf

[11] Brooks J Humanitarians under attack: tensions, disparities, and legal gaps in protection. Cambridge (MA): Harvard Humanitarian Initiative; 2015 Available from: http://hhi.harvard.edu/publications/humanitarians-under-attack-tensions-disparities-and-legal-gaps-protection

[12] Friedrich MJ Attacks on health increasing in conflict-ridden countries. JAMA. 2015;314(3):216 10.1001/jama.2015.7288

[13] Haar RJ Footer KH Singh S Sherman SG Branchini C Sclar J Measurement of attacks and interferences with health care in conflict: validation of an incident reporting tool for attacks on and interferences with health care in eastern Burma. Confl Health. 2014;8(1):23. 10.1186/1752-1505-8-23. 25400693 PMC4232629

[14] Human Rights Watch. Under attack: violence against health workers, patients and facilities. New York: Human Rights Watch; 2014 Co-published by the Safeguarding Health in Conflict Coalition Available from: http://www.safeguardinghealth.org/sites/shcc/files/under-attack.pdf

[15] International Committee of the Red Cross (ICRC) [Internet]. Respect for and protection of the personnel of humanitarian organizations. Geneva: ICRC; 1998 [cited 2015 Mar 6] Available from: https://www.icrc.org/eng/resources/documents/report/57jp85.htm

[16] International Committee of the Red Cross (ICRC) [Internet]. Geneva Convention IV relative to the protection of civilian persons in time of war. Geneva: ICRC; 1949 [cited 2016 Sep 29] Available from: https://ihl-databases.icrc.org/applic/ihl/ihl.nsf/INTRO/380

[17] Heisler M Baker E McKay D Attacks on health care in Syria—normalizing violations of medical neutrality? N Engl J Med. 2015;373(26):2489–2491. 10.1056/NEJMp1513512. 26580838 PMC4778548

[18] Healthcare in Danger Campaign [Internet] Geneva: International Committee of the Red Cross; International Red Cross and Red Crescent movement; 2015 Aug 26 [cited 2016 Sep 29] Available from: http://healthcareindanger.org/

[19] World Health Organization (WHO) [Internet]. Geneva: WHO; C 2016 Attacks on health care; [cited 2016 Sep 29] Available from: http://www.who.int/hac/techguidance/attacks_on_health_care/en/

[20] International Committee of the Red Cross (ICRC). Health care in danger: violent incidents affecting the delivery of health care, January 2012 to December 2014. Geneva: ICRC; 2015 Available from: https://www.icrc.org/en/publication/4237-health-care-danger-violent-incidents-affecting-delivery-health-care-january-2012

[21] Stoddard A Harmer A Di Domenico V Providing aid in insecure environments: 2009 update. Trends in violence against aid workers and the operational response. London: Overseas Development Institute; 2009 Available from: https://www.odi.org/sites/odi.org.uk/files/odi-assets/publications-opinion-files/4243.pdf

[22] St. George D Brown DL Anita Datar is the only known American killed in Mali. She was there to help. Washington Post [Internet] 2015 Nov 21 [cited 2016 Jan 31] Available from: https://www.washingtonpost.com/local/anita-datar-was-the-only-american-killed-in-mali-she-was-there-to-help/2015/11/20/70e0b0aa-8fe6-11e5-ae1f-af46b7df8483_story.html

[23] Hendrix S Jolted by terrorist attacks, U.S. travelers get serious about overseas trip security. Washington Post [Internet] 2016 Jan 14 [cited 2016 Jan 31] Available from: https://www.washingtonpost.com/local/jolted-by-terror-attacks-us-travelers-get-serious-about-overseas-trip-security/2016/01/14/b11e1bf2-b977-11e5-829c-26ffb874a18d_story.html

[24] Crump JA Sugarman J. Working Group on Ethics Guidelines for Global Health Training (WEIGHT). Ethics and best practice guidelines for training experiences in global health. Am J Trop Med Hyg. 2010;83(6):1178–1182. 10.4269/ajtmh.2010.10-0527. 21118918 PMC2990028

[25] Lumb A Murdoch-Eaton D Electives in undergraduate medical education: AMEE Guide No. 88. Med Teach. 2014;36(7):557–572. 10.3109/0142159X.2014.907887. 24787526

[26] Peace Corps [Internet]. Washington (DC): Peace Corps; [no date]. Safety & security; [cited 2016 Nov 4] Available from: https://www.peacecorps.gov/volunteer/health-and-safety/safety-and-security/

[27] InterAction [Internet]. Washington (DC): InterAction; C 2016 NGO security; [cited 2016 Sep 29] Available from: https://www.interaction.org/work/security

[28] InterAction. Minimum operating security standards and suggested guidance language. Washington (DC): InterAction; [2006?]. Available from: https://www.interaction.org/document/interaction-minimum-operating-security-standards-and-suggested-guidance-language

[29] Edwardson J Owens L Moran D Aluri J Kironji A Chen CCG Pre-departure preparation for international clinical work: a handbook. Int Urogynecol J Pelvic Floor Dysfunct. 2015;26(8):1111–1113. 10.1007/s00192-015-2682-0. 25994626

[30] Hansoti B Douglass K Tupesis J Runyon MS Sanson T Babcock C Guidelines for safety of trainees rotating abroad: consensus recommendations from the Global Emergency Medicine Academy of the Society for Academic Emergency Medicine, Council of Emergency Medicine Residency Directors, and the Emergency Medicine Residents. Acad Emerg Med. 2013;20(4):413–420. 10.1111/acem.12106. 23701352

[31] Butcher CA International health program: preventing health problems associated with living abroad. AAOHN J. 2004;52(2):77–85; quiz 86–87. 14979618

[32] Dell EM Varpio L Petrosoniak A Gajaria A McCarthy AE The ethics and safety of medical student global health electives. Int J Med Educ. 2014;5:63–72. 10.5116/ijme.5334.8051. 25341214 PMC4207171

[33] Tyagi S Corbett S Welfare M Safety on elective: a survey on safety advice and adverse events during electives. Clin Med (Northfield IL). 2006;6(2):154–156. 10.7861/clinmedicine.6-2-154. 16688972 PMC4953198

[34] Purkey E Hollaar G Developing consensus for postgraduate global health electives: definitions, pre-departure training and post-return debriefing. BMC Med Educ. 2016;16(1):159. 10.1186/s12909-016-0675-4. 27259965 PMC4893221

[35] de Cortina SH Arora G Wells T Hoffman RM Evaluation of a structured predeparture orientation at the David Geffen School of Medicine’s Global Health Education Programs. Am J Trop Med Hyg. 2016;94(3):563–567. 10.4269/ajtmh.15-0553. 26755562 PMC4775891

[36] Wells T Arora T Evaluation of a structured pre-departure orientation in a medical student global health education program. Ann Glob Health. 2015;81(1):177 10.1016/j.aogh.2015.02.904

[37] Wille C Fast L Aid, Gender and security: the gendered nature of security events affecting aid workers and aid delivery. Bellevue (Switzerland): Insecurity Insight; 2011 Available from: http://www.insecurityinsight.org/files/Security%20Facts%202%20Gender.pdf

[38] Gaul A Keegan M Lawrence M Lyra Ramos M NGO security: does gender matter? London: Save the Children; 2006 Available from: http://www.alnap.org/resource/12068

[39] Fast L Wiest D Security perceptions survey. Notre Dame (IN): Kroc Institute for International Peace Studies, University of Notre Dame; 2007 Available from: https://www.eisf.eu/wp-content/uploads/2014/09/0009-Fast-Wiest-2007-Security-Perceptions-Survey10.pdf

[40] United Nations Office for Disaster Risk Reduction (UNISDR) [Internet]. Geneva: UNISDR. Terminology on DRR; 2007 [updated 2009; cited 2016 Sep 25] Available from: https://www.unisdr.org/we/inform/terminology

[41] Federal Emergency Management Agency (FEMA). Developing and maintaining emergency operations plans: comprehensive preparedness guide 101. Washington (DC): FEMA; 2010 Available from: https://www.fema.gov/pdf/about/divisions/npd/CPG_101_V2.pdf

